# MEANINGFUL ENGAGEMENT AND QUALITY OF LIFE AMONG ASSISTED LIVING RESIDENTS WITH DEMENTIA: EMERGENT FINDINGS

**DOI:** 10.1093/geroni/igz038.3430

**Published:** 2019-11-08

**Authors:** Joy Ciofi, Candace L Kemp, Alexis A Bender, Elisabeth O Burgess, Jennifer C Morgan, Fayron Epps, Patrick Doyle, Molly M Perkins

**Affiliations:** 1 Georgia State University, Atlanta, Georgia, United States; 2 Emory University, Atlanta, Georgia, United States; 3 Gerontology Institute, Georgia State University, Atlanta, Georgia, United States; 4 Center for Innovative Care in Aging, Johns Hopkins School of Nursing, Baltimore, Maryland, United States; 5 Division of General Medicine and Geriatrics, Emory University School of Medicine, Atlanta, Georgia, United States

## Abstract

This poster provides an overview of the aims, methods, and emergent findings from an ongoing five-year NIA-funded project (R01AG062310) examining meaningful engagement and quality of life among assisted living (AL) residents with dementia. The overall goal of this project is to determine how opportunities for meaningful engagement can best be recognized, created, and maintained for individuals with different dementia types and varying levels of functional ability. Guided by grounded theory, this qualitative study will involve 12 diverse AL communities in and around Atlanta, Georgia, USA. Presently, our interdisciplinary team is collecting data in four communities using ethnographic observations, semi-structured interviews, and resident record review. We are studying daily life in each community, following 30 resident participants, and actively recruiting and interviewing their formal and informal care partners. Based on ongoing analysis, we offer key emergent findings. First, meaningful engagement is highly individualized and dynamic. Differing personal interests, along with wide variations in cognitive and physical abilities, can present challenges for AL community staff and other care partners when trying to recognize what constitutes meaningful engagement for residents. Second, multiple complex factors interplay to shape the experience of meaningful engagement among persons living with dementia, such as personal characteristics, care partner background and training, AL community design and philosophy, and state/corporate regulations. Finally, flexibility and ‘meeting the resident where they are at’ appear to be critical to identifying and fostering meaningful engagement for persons living with dementia. We discuss the implications of these preliminary findings for translation, dissemination, and future research.

